# Tailoring of physical properties of RF-sputtered ZnTe films: role of substrate temperature

**DOI:** 10.3762/bjnano.16.25

**Published:** 2025-03-05

**Authors:** Kafi Devi, Usha Rani, Arun Kumar, Divya Gupta, Sanjeev Aggarwal

**Affiliations:** 1 Ion Beam Centre, Department of Physics, Kurukshetra University, Kurukshetra-136119, India

**Keywords:** bandgap, physical properties, RF sputtering, substrate temperature, ZnTe

## Abstract

In this study, zinc telluride (ZnTe) films were grown on quartz substrates at room temperature, 300 °C, 400 °C, 500 °C, and 600 °C using RF sputtering. The thickness of the films has been found to decrease from 940 nm at room temperature to 200 nm at 600 °C with increasing substrate temperature. The structural investigation using grazing incidence angle X-ray diffraction revealed that films deposited at room temperature are amorphous; those deposited at other substrate temperatures are polycrystalline with a cubic zincblende structure and a preferred orientation along the [111] direction. An increase in crystallite size (from 37.60 ± 0.42 Å to 68.88 ± 1.04 Å) is observed with increased substrate temperature. This leads to a reduction in microstrain and dislocation density. The optical studies using UV–vis–NIR spectroscopy reveal that the transmittance of films increases with substrate temperature. Further, the shift in transmittance threshold towards lower wavelengths with substrate temperature indicates that the optical bandgap of the films can be tuned from 1.47 ± 0.02 eV to 3.11 ± 0.14 eV. The surface morphology of the films studied using atomic force microscopy reveals that there is uniform grain growth on the surface. Various morphological parameters such as roughness, particle size, particle density, skewness, and kurtosis were determined. Current–voltage characteristics indicate that the conductivity of the films increased with substrate temperature. The observed variations in structural, morphological, and optical parameters have been discussed and correlated. The wide bandgap (3.11 eV), high crystallinity, high transmittance, and high conductivity of the ZnTe film produced at 600 °C make it a suitable candidate for use as a buffer layer in solar cell applications.

## Introduction

The industrialization and burning of fossil fuels to fulfil the growing demands of energy results in environmental pollution. Environmentally friendly resources such as solar and wind energy can act as a substitute for these non-renewable energy resources because of their sustainability and abundance. Commonly, silicon-based solar cells are used for this purpose. However, the requirement of very pure material for making these devices leads to high production cost. Metal chalcogenide-based solar cells, because of their low cost and comparable efficiency, can act as a substitute for the Si-based technology. Metal chalcogenide (II–VI) compounds have numerous applications in optoelectronic devices such as light-emitting diodes [1], display devices [2], infrared detectors [3], and terahertz emitters [4]. Owing to their suitable physical properties (deposition at low temperatures and good thermal stability) and unique optical properties due to quantum confinement size effects, metal chalcogenides are of importance in different technological domains. Metal chalcogen compounds are composed of a transition metal with one or more members of the chalcogen family, and they exhibit semiconducting properties.

Zinc telluride (ZnTe) is a binary II–VI semiconductor with a direct bandgap of 2.26 eV, which lies in the visible range of the electromagnetic spectrum. ZnTe is a p-type semiconductor because of zinc vacancies and has a low electron affinity of 3.53 eV at room temperature [5]. It exists in both zincblende and wurtzite structures, depending on the deposition method and deposition parameters. ZnTe is sensitive towards visible and infrared illumination; hence, it is used in the fabrication of optoelectronic devices and infrared detectors. Moreover, its electrical aspects are alterable in intrinsic or doped binary (ZnTe) and ternary (CdZnTe) compounds.

Currently many efforts are made to increase the efficiency of CdTe-based solar cells. A maximum efficiency of 22.1% has been achieved using CdTe-based solar cells. The efficiency can be tuned by the formation of a stable ohmic back contact. For this, a material with a bandgap greater than 5.78 eV, that is, the sum of CdTe electron affinity (4.28 eV) and bandgap (1.5 eV), would be required. Such a material is not available; therefore, the formation of a Schottky barrier is unavoidable. Because of the small 0.1 eV valance band offset at the CdTe/ZnTe interface, which is best for carrier transport, ZnTe can be used as a buffer layer in CdTe-based solar cells for back contact. Moreover, n-type zinc telluride films can be used in the window layer as a substitute for CdS [6].

Zinc telluride films are highly resistive with a resistivity of about several megaohm·centimetres [7]. The resistivity of the films depends on the structure, grain boundary defects, and surface morphology of the films. These properties can be altered by varying the deposition method as well as the deposition parameters. In literature, there are several reports of zinc telluride films deposited using various physical and chemical methods such as molecular beam epitaxy [8], electron-beam evaporation [9], thermal evaporation [10], pulsed laser deposition (PLD) [11], and RF sputtering [12]. RF sputtering is a versatile technique because various process parameters such as RF power, deposition time, substrate–target distance, substrate temperature, and pressure during deposition inside the chamber can be varied. These process parameters have a remarkable impact on the structural, optical, and electrical properties of the grown films. Further, films with uniform thickness can be grown using this technique.

Bellakhder et al. [13] have investigated the impact of varying RF power on the structure, optical, and electrical properties of RF-sputtered ZnTe films and found that the deposited films are highly resistive and have low refractive index because of the polycrystalline nature of films. Isik et al. [14] carried out studies on the structure and temperature-dependent optical properties of magnetron-sputtered ZnTe films. Bacaksiz et al. [15] reported the effect of substrate temperature (−123 and 27 °C) on structural, morphological, optical, and electrical properties of ZnTe films deposited by evaporation. Rakhshani et al. [16] reported the impact of substrate temperature (35 and 305 °C), thermal annealing, and nitrogen doping on optoelectronic properties of ZnTe films and established an optimal doping concentration of nitrogen for lowering the resistivity of the grown films. Further, there are reports [17–23] which show that chemical composition and morphology of the substrate affect the properties of grown films. Therefore, a suitable substrate needs to selected before the deposition of films. The thermal expansion coefficient of substrate and the lattice mismatch between film and substrate are two important parameters for substrate selection. To the best of our knowledge, studies related to the impact of the substrate (silicon and quartz) and film deposition temperature up to 600 °C on the structural, morphological, optical, and electrical behaviour of RF-sputtered ZnTe films are very rare. Therefore, it is necessary to carry out a detailed study in this regard and to find out the optimum parameters for film deposition for applications in optoelectronic devices.

Recently we reported the impact of substrate temperature on the structure, morphology, and reflectance behaviour of ZnTe films on silicon substrates [24]. It was observed that 400 °C is the optimum temperature for the growth of ZnTe films.

In this study, our we focus on optimizing the deposition parameters for ZnTe films on quartz substrates and study the impact of substrate temperature (300–600 °C) on various physical properties (structure, morphology, optical and electrical properties, and luminescence) of RF-sputtered ZnTe films. Quartz is an import substrate because of its high transparency, high melting point, and low thermal expansion coefficient. This study helps in optimizing the substrate temperature to grow films with superior quality in terms of grain size, dislocation density, and optical and electrical properties to enhance the performance of ZnTe-based optoelectronic devices and solar cells.

## Experimental

ZnTe films were deposited on quartz (Qz) using RF sputtering at different substrate temperatures. Prior to film deposition, the substrates were cleansed in an ultrasonic cleaner using acetone and isopropyl alcohol sequentially for 10 min at room temperature. The cleaned substrates were then dried in air and placed on the substrate holder in the chamber. A ZnTe target (dimensions 2 inch diameter and 3 mm thickness) having 99.99% purity was used for sputter deposition of the films. The substrate was kept at a distance of 7 cm from the target. The different deposition parameters are specified in Table 1.

**Table 1:** Deposition parameters for film fabrication.

Deposition parameters	Value

RF power	60 W
base pressure	10^−5^ mbar
working pressure	10^−3^ mbar
deposition environment	Ar gas
Ar gas flow	10 sccm
substrate temperature	room temperature (R.T.), 300 °C, 400 °C, 500 °C, and 600 °C
deposition time	30 min

The thickness of the fabricated ZnTe/Qz films was determined using spectroscopic ellipsometry (SE). The experimental parameters ψ and Δ were recorded at an incident angle of 70° with respect to film surface using a SENTECH ellipsometer in the wavelength range of 200–1000 nm. The thickness of the films was found to be 940 ± 0.53 nm, 623 ± 0.16 nm, 563 ± 0.02 nm, 337 ± 0.02 nm, and 200 ± 0.30 nm for the films deposited at room temperature, 300 °C, 400 °C, 500 °C, and 600 °C, respectively.

The structural aspects of the ZnTe/Qz films were analysed using grazing incidence X-ray diffraction (GXRD) on a Bruker AXS D8 Advance with Cu Kα radiation (λ = 1.5406 Å) available at Ion Beam Centre, Kurukshetra University. The grazing incidence angle was fixed at 0.5°. The diffraction pattern was recorded in the 2θ range of 20°–70° with a step increment of 0.07°.

The optical properties of ZnTe/Qz films were analysed from transmittance spectra obtained using a Shimadzu UV–vis–NIR spectrophotometer (UV-3600 Plus) equipped with Integrating Sphere Assembly (Model-ISR-603) in the wavelength range of 200–2000 nm (accuracy 1 Å) available at Ion Beam Centre, Kurukshetra University.

The photoluminescence (PL) emission spectra of ZnTe/Qz films were recorded using a HORIBA Scientific (Fluorescence 3.5) spectrophotometer under 320 nm excitation produced by a xenon arc lamp.

For investigating the surface topography, atomic force microscopy (AFM) micrographs of ZnTe/Qz films were recorded (scan area 2 × 2 µm^2^) using a Bruker multimode-8 AFM in the ScanAsyst mode at the Ion Beam Centre, Kurukshetra University. The obtained micrographs were then analysed regarding various statistical parameters such as roughness, skewness, kurtosis, and power spectral density using the NanoScope Analysis software.

Surface morphology and composition of the films were studied by field-emission scanning electron microscopy attached with energy-dispersive X-ray spectroscopy (EDS) operated at 10 keV.

The current–voltage (*I*–*V*) characteristics of the films were measured in the voltage range from −1 V to 1 V using a two-probe Keithley 4200 A-SCS parametric analyser available at Ion Beam Centre, Kurukshetra University.

## Results and Discussion

### X-ray diffraction studies

**Figure 1:**
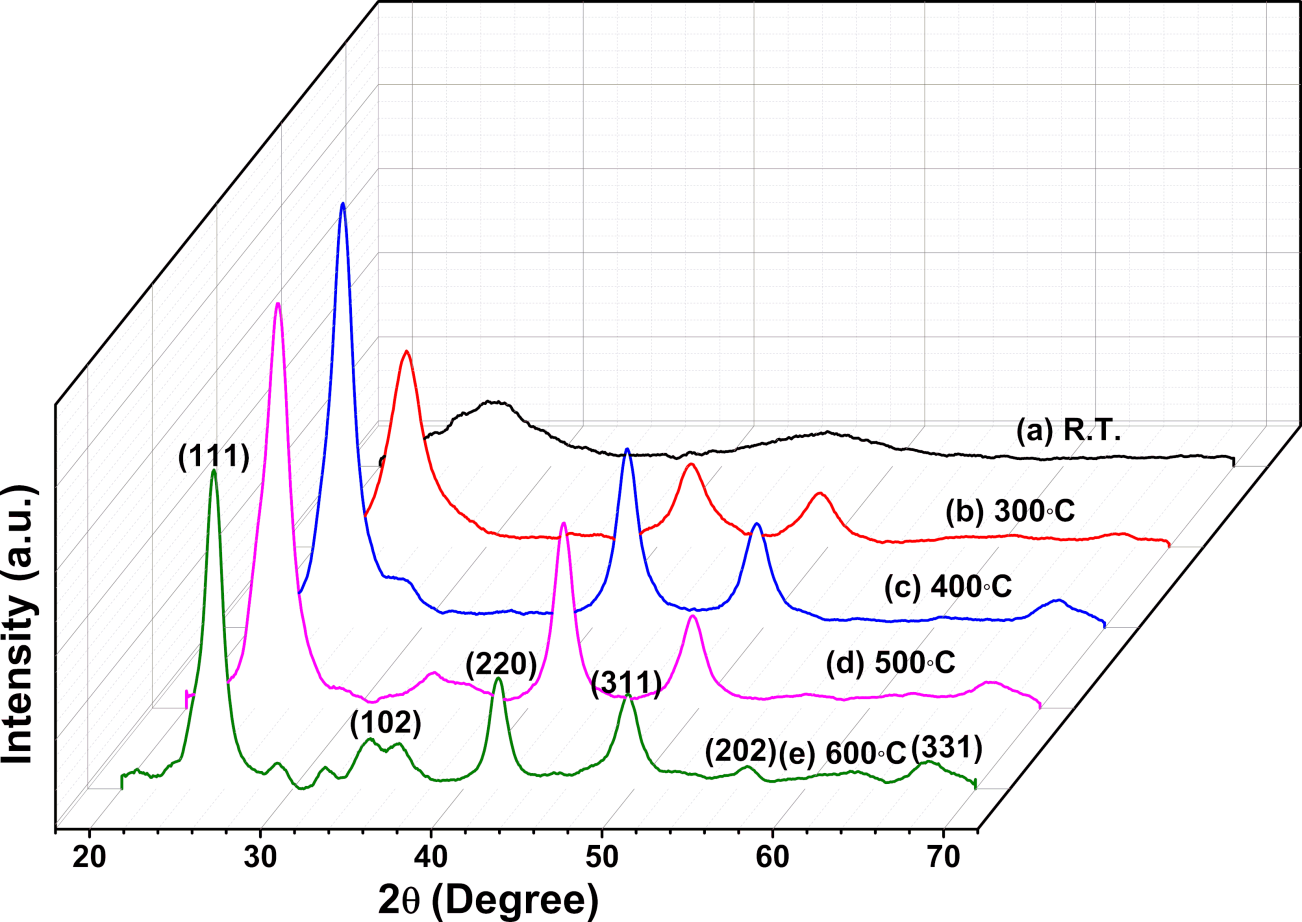
GXRD pattern of the ZnTe/Qz films deposited at room temperature and different substrate temperatures in the range of 300–600 °C.

GXRD patterns of ZnTe films grown on quartz substrates at different substrate temperatures (R.T.–600 °C) are presented in Figure 1. A broad hump in the GXRD pattern of the film deposited at room temperature indicates that the film is amorphous. The three diffraction peaks in the GXRD pattern at 2θ = 25.33°, 42.04°, and 49.47° correspond to (111), (220), and (311) reflection planes, respectively. They are observed for all films deposited above room temperature. Another diffraction peak at 2θ = 67.17°, corresponding to (331) reflection planes, starts evolving at a substrate temperature of 400 °C. Indexing of peaks and comparison with JCPDS data (card no. 01-071-8947) reveals that the deposited films are polycrystalline with a cubic zincblende structure. At a substrate temperature of 600 °C, some other diffraction peaks at 2θ = 34.34° and 56.52° are observed. Comparison with the JCPDS card no. 00-019-1482 reveals that these correspond, respectively, to the (102) and (202) reflections of the hexagonal ZnTe phase. Thus, a mixture of phases exists at this substrate temperature. Other very low-intensity peaks at this temperature are from pure Zn and Te.

The deposition temperature affects the preferred crystallite orientation. To quantitatively analyse the impact of deposition temperature on the preferred orientation, the texture coefficient (TC(hkl)) was determined using the relation [25]


[1] $$\text{TC}\left(\text{hkl}\right)=\frac{\frac{I(\text{hkl})}{I_{\text{S}}(\text{hkl})}}{\frac{1}{n}\sum \frac{I(\text{hkl})}{I_{\text{S}}(\text{hkl})}},$$

where *I*(hkl) stands for the Lorentz-fitted peak intensity, *I*_s_(hkl) represents the intensity of peaks in the referenced JCPDS database at corresponding angles, and *n* represents the number of peaks in the diffraction pattern. The calculated values of TC(hkl) for different diffraction planes is given in Table 2.

**Table 2:** The structural parameters crystallite size (*D*), dislocation density (Δ), microstrain (ε), interplanar spacing (*d*_hkl_), lattice constant (*a*), and texture coefficient (TC) of ZnTe films fabricated at different substrate temperatures.

Substrate temperature (°C)	2θ (°)	(hkl)	Crystallite size (*D*)	Δ (×10^17^ m^−2^)	ε (×10^−2^)	*d*_hkl_ (Å)	*a* (Å)	TC

R.T.	—	—	—	—	—	—	—	—
300	25.33 42.04 49.47	(111) (220) (311)	37.60 ± 0.42	0.7	4.3	3.511	6.08	1.28 0.83 0.88
400	25.34 42.05 49.61	(111) (220) (311)	54.26 ± 0.60	0.3	3.0	3.510	6.08	1.32 0.87 0.80
500	25.30 42.08 49.75	(111) (220) (311)	52.11 ± 1.10	0.36	3.1	3.516	6.09	1.32 0.96 0.72
600	25.35 42.04 49.61	(111) (220) (311)	68.88 ± 1.04	0.21	2.4	3.509	6.07	1.34 0.74 0.91

TC values greater than or equal to one indicate the preferential orientation in that particular (hkl) direction, that is, more crystallites grow in this particular direction, while TC values less than one indicate that the orientation is random. The TC value of the (111) planes is greater than one, which indicates that [111] is the preferred orientation for all ZnT/Qz film samples. The TC value for the (111) planes increases from 1.16 to 1.34 with the rise of substrate temperature. Other preferred orientations are [220] and [311], but with a smaller degree of texture compared to the [111] direction. The texture coefficient value for the (220) planes increases from 0.83 to 0.96 and decreases for the (311) planes from 0.88 to 0.72 with the rise of substrate temperature (300–500 °C). At a substrate temperature of 600 °C, the TC value for the (311) planes is higher than that for the (220) planes. Further, some other low-intensity peaks corresponding to (102) and (202) planes are also observed at this temperature, which indicates that, at higher substrate temperatures, the preferred orientation and structure may change.

The higher texture coefficient of the (111) planes indicates a minimum surface energy density of these planes because crystal growth in films occurs in the direction of the lowest surface energy. The intensity of X-rays in a diffraction pattern is a function of the atomic structure factor. The change in the texture coefficient of different planes shows that the atomic densities of the planes change with the substrate temperature.

The increase in intensity for the most preferred orientation along the [111] direction is observed with rising substrate temperature up to 400 °C. This may be because atoms have more thermal energy with increasing substrate temperature; therefore, the surface mobility of atoms increases, which leads to rearrangement and the increase in intensity. However, a decrement in intensity is observed for the films deposited at higher substrate temperatures of 500 and 600 °C. This may be because, at rising substrate temperatures, the chance for dissociation and desorption of atoms increases, which causes a decrease in the intensity [26]. To investigate the effect of substrate temperature on peak broadening of ZnTe films, various structural parameters including crystallite size, microstrain, and dislocation density were calculated corresponding to the most prominent peak.

The crystallite size (*D*) was calculated using Scherrer’s formula [27]


[2] $$D=\frac{0.94\lambda }{\beta \cos\theta },$$

where λ = 1.5406 Å is the X-ray wavelength and β denotes the full width at half maximum (in radians).

The microstrain (ε) is calculated using the formula [27]


[3] $$\varepsilon =\frac{\beta }{4\tan\theta }.$$

The dislocation density (Δ) is calculated using the formula [27]


[4] $$\Delta =\frac{1}{D^{2}},$$

where *D* represents the crystallite size.

The calculated values of *D*, ε, and Δ are listed in Table 2. Films deposited at R.T. are amorphous; therefore, the crystallite size has not been calculated. The crystallite size increases from 37.60 to 54.26 Å with the rise in substrate temperature from 300 to 400 °C. There is no appreciable change in crystallite size at 500 °C. While a significant increase in crystallite size from 52.11 Å at 500 °C to 68.66 Å at 600 °C is observed. All these alterations in crystallite size can be explained in terms of growth processes occurring during film fabrication. In RF sputtering, the film formation is preceded by three steps, namely, condensation, nucleation, and crystallization on the substrate surface. The mobility of atoms on the substrate surface is very much affected by the substrate temperature. At low substrate temperatures, because of the low diffusion rate and low mobility of atoms, columnar microstructures form on the substrate surface. With the increase in substrate temperature, mobility and diffusion rate of atoms increase, which results in the evolution of grains that further recrystallize at higher substrate temperatures [28]. The observed variation in the crystallite size is due to changes in mobility and diffusion rate of atoms with substrate temperature.

The interplanar spacing (*d*_hkl_) and lattice constant (*a*) were calculated for the (111) plane using Bragg’s law [29],


[5] $$d_{\text{hkl}}=\frac{n\lambda }{2\sin\theta },$$

[6] $$a=d_{\text{hkl}}\sqrt{h^{2}+k^{2}+l^{2}},$$

where θ represents the Bragg angle, λ is the X-ray wavelength, *d* is the interplanar spacing, and *a* is the lattice constant for a particular (hkl) plane. The calculated values are presented in Table 2. The lattice constant value for ZnTe/Qz films is slightly larger than in the bulk (6.07 Å, JCPDS card no.01-071-8947). This may be on account of lattice mismatch or difference in thermal expansion coefficient between film and substrate, which ultimately lead to the development of stress and strain within the film. At a substrate temperature of 600 °C, the lattice constant value is the same as in the bulk material. The strain in films occurs due to lattice mismatch between film and bulk. The microstrain in films was calculated using Equation 3. The microstrain decreases with increasing substrate temperature. This implies that imperfections along grain boundaries decrease with temperature. The dislocation density, which is defined as the average number of dislocations present per unit volume in a crystal was calculated using Equation 4, and it decreases with increasing substrate temperature. The high value of crystallinity and low value of microstrain and dislocation density at 600 °C shows that good quality films can be fabricated at this temperature.

### Morphological investigation

**Figure 2:**
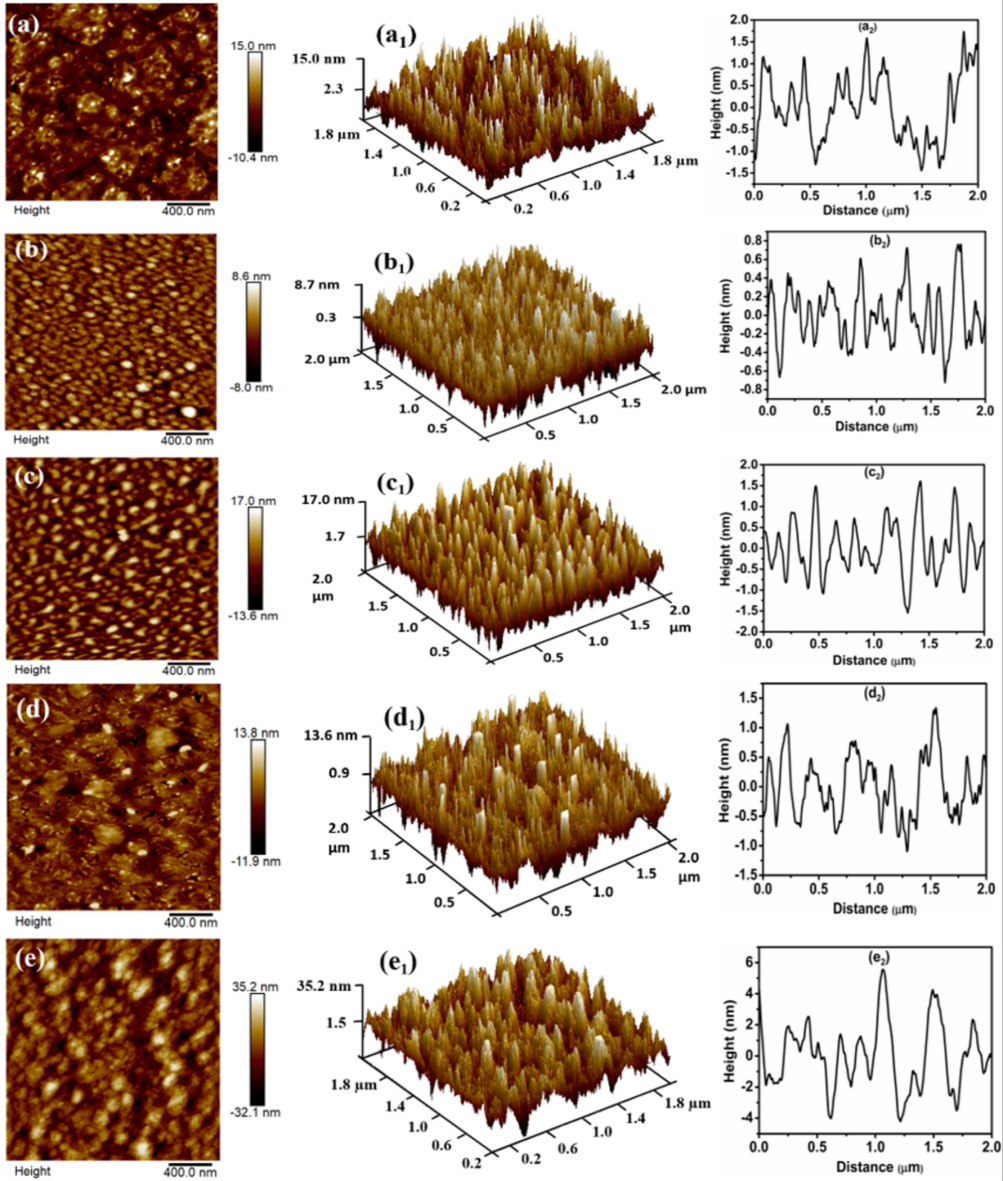
2D (a–e) and 3D (a_1_–e_1_) AFM images and Height profile (a_2_–e_2_) analysis of ZnTe films deposited at (a, a_1_, a_2_) R.T. and substrate temperatures of (b, b_1_, b_2_) 300 °C, (c, c_1_, c_2_) 400 °C, (d, d_1_, d_2_) 500 °C, and (e, e_1_, e_2_) 600 °C.

AFM was utilized to study the evolution of surface morphology of ZnTe/Qz films grown at different substrate temperatures. 2D and 3D AFM images (scan area 2 × 2 µm^2^) are presented in Figure 2. The 2D images (Figure 2a–e) show that the surface of all film samples is covered with densely packed spherical nanograins. The 3D images (Figure 2a_1_–e_1_) reveal a columnar growth on the surface of the films. The AFM micrographs were analysed using NanoScope software, and various morphological parameters including roughness, particle density, particle size, skewness, and kurtosis were summarized in Table 3. To investigate the quality of the surface and the texture of the films, the root mean square roughness (*R*_q_) was measured. *R*_q_ for the ZnTe film produced at R.T. is 3.49 nm. It increases from 2.27 nm at 300 °C to 3.53 nm at 400 °C. There is no appreciable change in roughness at a substrate temperature of 500 °C (i.e., it lies within the error of the value at 400 °C). However, a significant increase in roughness from 3.12 nm at 500 °C to 9.28 nm at 600 °C was observed. These results are in correlation with our GXRD results, where a similar change in crystallinity was observed with substrate temperature [30]. The surface growth of films can be investigated in terms of two parameters, namely, height fluctuation and lateral aggregation among the spherical nanograins. The height profile for ZnTe films fabricated at various substrate temperatures is given in Figure 2a_2_–e_2_. The height fluctuation is related to the number density of particles, and the lateral aggregation is related to the particle size [31].

**Table 3:** Variation in surface parameters (i.e., skewness, kurtosis, roughness, density, and particle size) with substrate temperature.

Substrate temperature (°C)	Roughness (nm)	Particle density (µm^−2^)	Particle size (nm)	Skew-ness	Kurtosis	Power exponent (*b*)

R.T.	3.49 ± 0.27	37.43	59.03 ± 1.74	0.94	4.64	2.20
300	2.27 ± 0.24	82.22	54.72 ± 1.54	0.19	3.29	1.69
400	3.53 ± 0.23	46.76	58.88 ± 0.70	0.54	3.41	1.60
500	3.12 ± 0.13	45.24	63.58 ± 1.06	0.25	3.91	1.84
600	9.28 ± 0.81	27.13	83.99 ± 1.37	0.15	3.02	1.12

Figure 2a_2_–e_2_ shows that the height fluctuation first increases from R.T. to 300 °C and then continuously decreases with substrate temperature (300–600 °C). This indicates that the particle density first increases and then continuously decreases with substrate temperature accordingly. The lateral aggregation on the surface is inversely related to the height fluctuation. Thus, lateral aggregation (i.e., particle size) first decreases on rising the substrate temperature from R.T. to 300 °C and then continuously increases with substrate temperature (300–600 °C).

The symmetry of the surface of grown films can be investigated in terms of skewness and kurtosis. A zero value of skewness shows that the surface is symmetric, that is, a flat surface. Positive skewness values indicate that peaks are dominant, and negative skewness values indicate that valleys are dominant. The distribution of height on the surface is studied in terms of kurtosis. A kurtosis value equal to three means that the height distribution on the surface is Gaussian, and the surface is called mesokurtic. A kurtosis value of less than three points toward a platykurtic surface, and a kurtosis value greater than three indicates that peaks are dominant. It can be seen from Table 3 that the skewness value is positive and the kurtosis value is greater than three. This shows that peaks are dominant over valleys. This is also evident from the 3D micrographs (Figure 2a_2_–e_2_).

**Figure 3:**
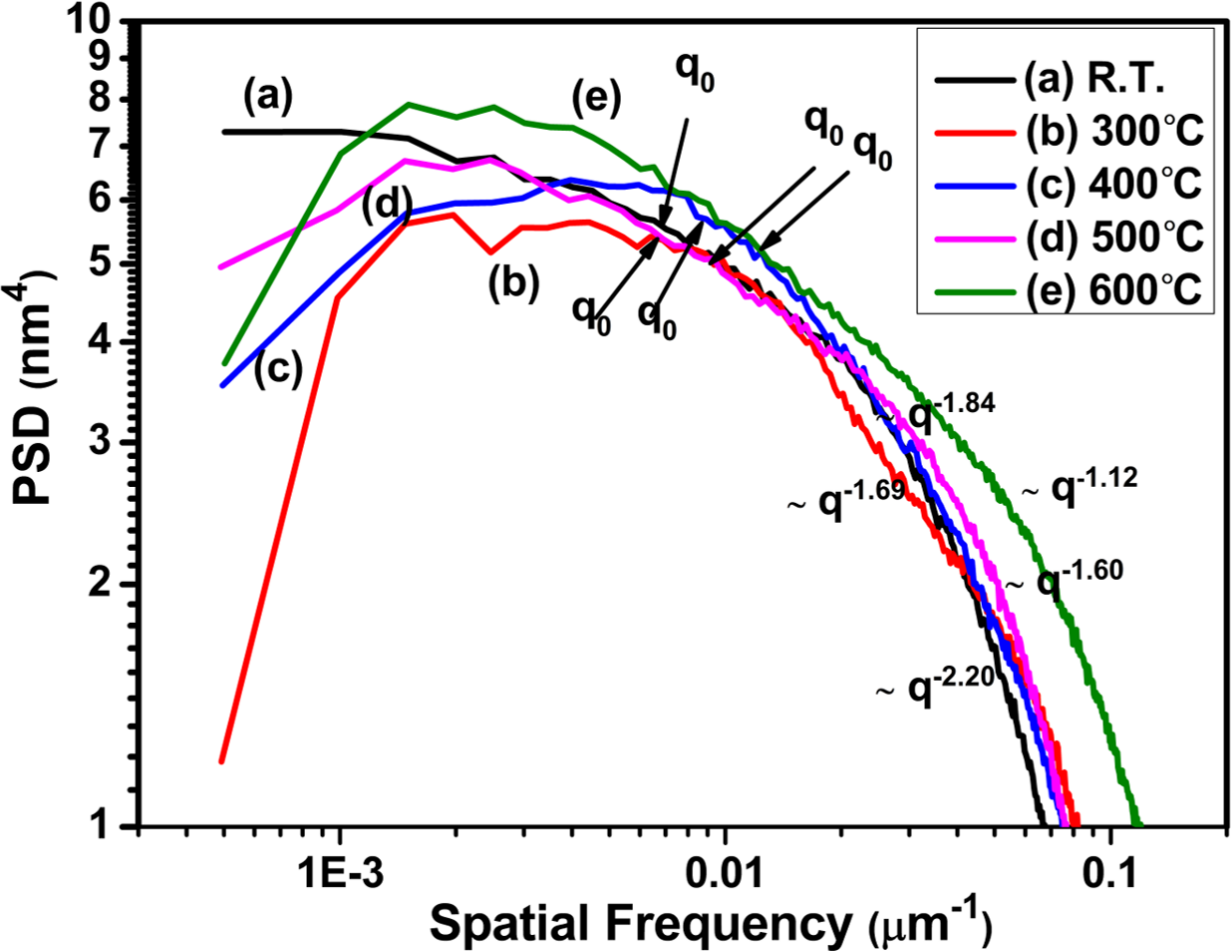
Power spectral density (PSD)-vs-frequency curve of ZnTe films produced at (a) R.T. and substrate temperatures of (b) 300 °C, (c) 400 °C, (d) 500 °C, and (e) 600°C.

The *R*_q_ value only gives information about the vertical fluctuation in height on a surface. To get more insight into possible surface growth mechanisms and variations in surface roughness, both vertically and laterally power spectral density (PSD) analyses were carried out. In PSD analysis, the surface is divided into various spatial wavelengths, and a comparative study of roughness is done over different frequency ranges. Figure 3 depicts the log–log plot for the evolution of PSD with spatial frequency (*q*) for ZnTe/QZ films deposited at different substrate temperatures. The information about surface corrugation is obtained from the slope of the line connecting two points on the surface. The surface corrugation is small for points separated by a length larger than the correlation length (η = 1/*q*_0_). As a result, for *q* < *q*_o_, the PSD is independent of frequency, and the surface is considered to be flat in this region. But for *q* > *q*_0_, surface corrugation is significant, and the PSD is found to decrease with spatial frequency. The points where a sharp decrease in the PSD curve takes place are indicated in Figure 3 by vertical arrows with values of *q*_0_. The integral of the PSD in the entire frequency range is proportional to the surface roughness. Therefore, PSD curves corresponding to higher roughness should lie at higher ordinates compared to PSD curves for lower roughness. In our case, it was found that the films fabricated at 300 and 600 °C had the lowest and highest roughness, respectively. Therefore, the PSD curves for films fabricated at 300 and 600 °C are at the lowest and highest ordinates, respectively. The PSD curve follows a power law as per equation


[7] $$\text{PSD}=Aq^{-b},$$

where *A* is a constant and *b* is the power law exponent. The value of *b* gives an idea about the film growth mechanism that is accountable for morphological changes on the surface of ZnTe films grown on quartz substrate at different substrate temperatures. Values of *b* equal to 1, 2, 3, and 4 represent four surface growth mechanisms, that is, viscous flow, evaporation–condensation, volume diffusion, and surface diffusion, respectively. The experimental data was fitted using Equation 7 in the high-frequency region, and the obtained values of *b* are listed in Table 3. For the film deposited at R.T., the value of *b* lies between 2 and 3, which implies that the possible surface growth mechanism is a combined effect of evaporation–condensation and volume diffusion. The *b* value for the films deposited at substrate temperature of 300–600 °C lies in the range from 1.12 to 1.84. This shows that the possible surface growth mechanism at these temperatures is a combined effect of viscous flow and evaporation–condensation [32].

**Figure 4:**
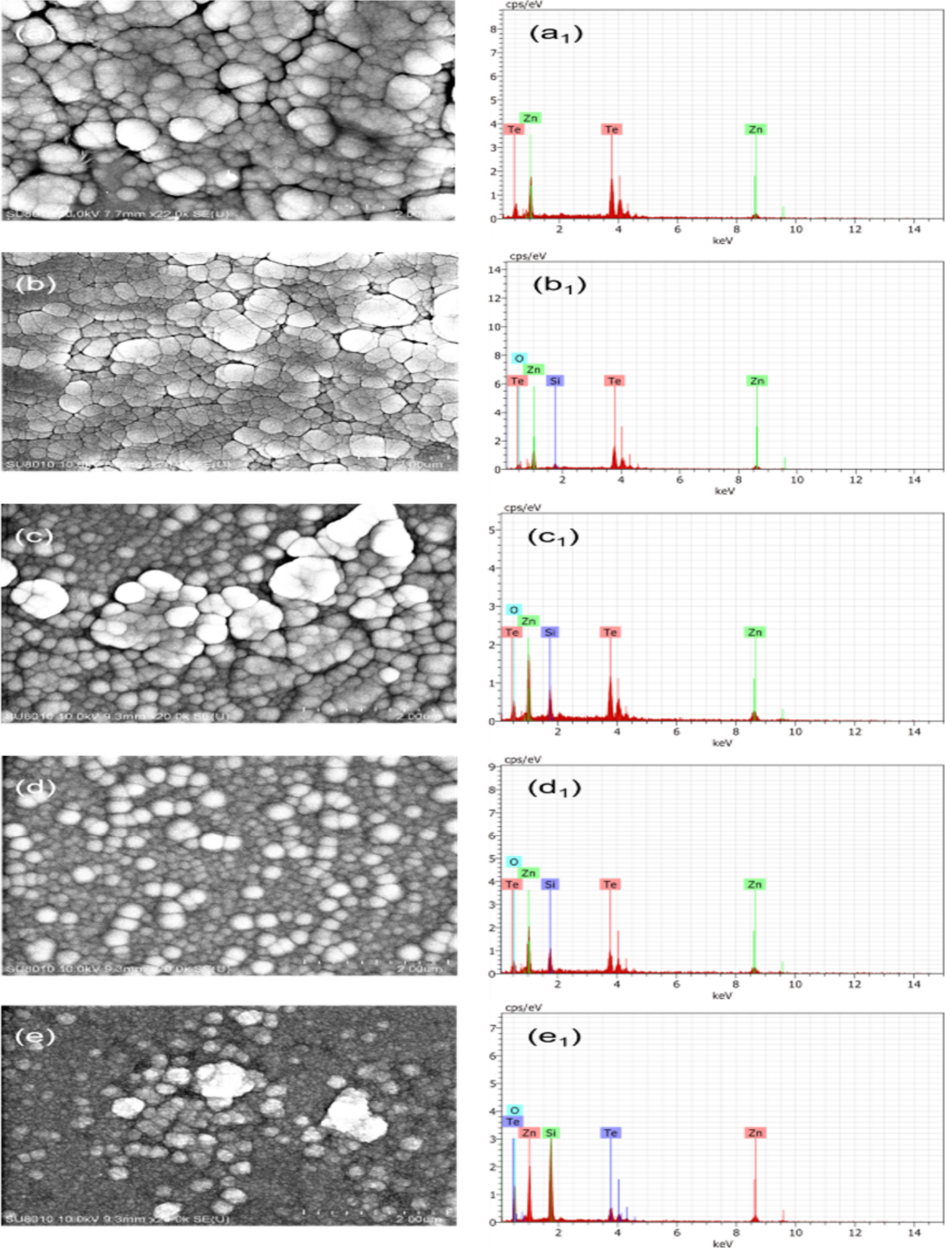
FESEM micrographs (a–e) and EDS spectra (a_1_–e_1_) of ZnTe films deposited at (a, a_1_) R.T., (b, b_1_) 300 °C, (c, c_1_) 400 °C, (d, d_1_) 500 °C, and (e, e_1_) 600 °C. (The scale bars are 2 µm).

The microstructure of ZnTe films deposited at different substrate temperatures has been studied using FESEM. The FESEM micrographs are presented in Figure 4a–e, along with the EDS results (Figure 4a_1_–e_1_). The films consist of spherical well-connected particles that are uniformly distributed over the entire surface of the substrate. A significant change in particle size along with a change in agglomerated particle density occurs with increasing substrate temperature. The surface is free from pinholes and voids, and clusters of particles can be seen. Quantitative analysis of the surface composition was carried out using EDS, and the obtained spectra are shown in Figure 4a_1_–e_1_. The existence of zinc and tellurium peaks indicates the formation of ZnTe thin films. The silicon and oxygen peaks are from the quartz substrate. The atomic percentages of Zn and Te are listed in Table 4. The film deposited at room temperature (R.T.) is of stoichiometric ZnTe. The elemental composition of the films changes with increasing substrate temperature. This variation in the atomic percentage of different elements may be due to the difference in the vapour pressures of Zn and Te.

**Table 4:** Elemental composition of ZnTe films obtained from EDS.

Element	R.T.	300 °C	400 °C	500 °C	600 °C

Zn (atom %)	50.47	45.14	43.54	44.02	22.58
Te (atom %)	49.53	36.33	22.09	17.65	5.43
Si (atom %)	—	7.22	15.84	17.83	32.45
O (atom %)	—	11.31	18.54	20.50	39.54

### Optical properties

#### Transmittance, absorbance, and optical bandgap

**Figure 5:**
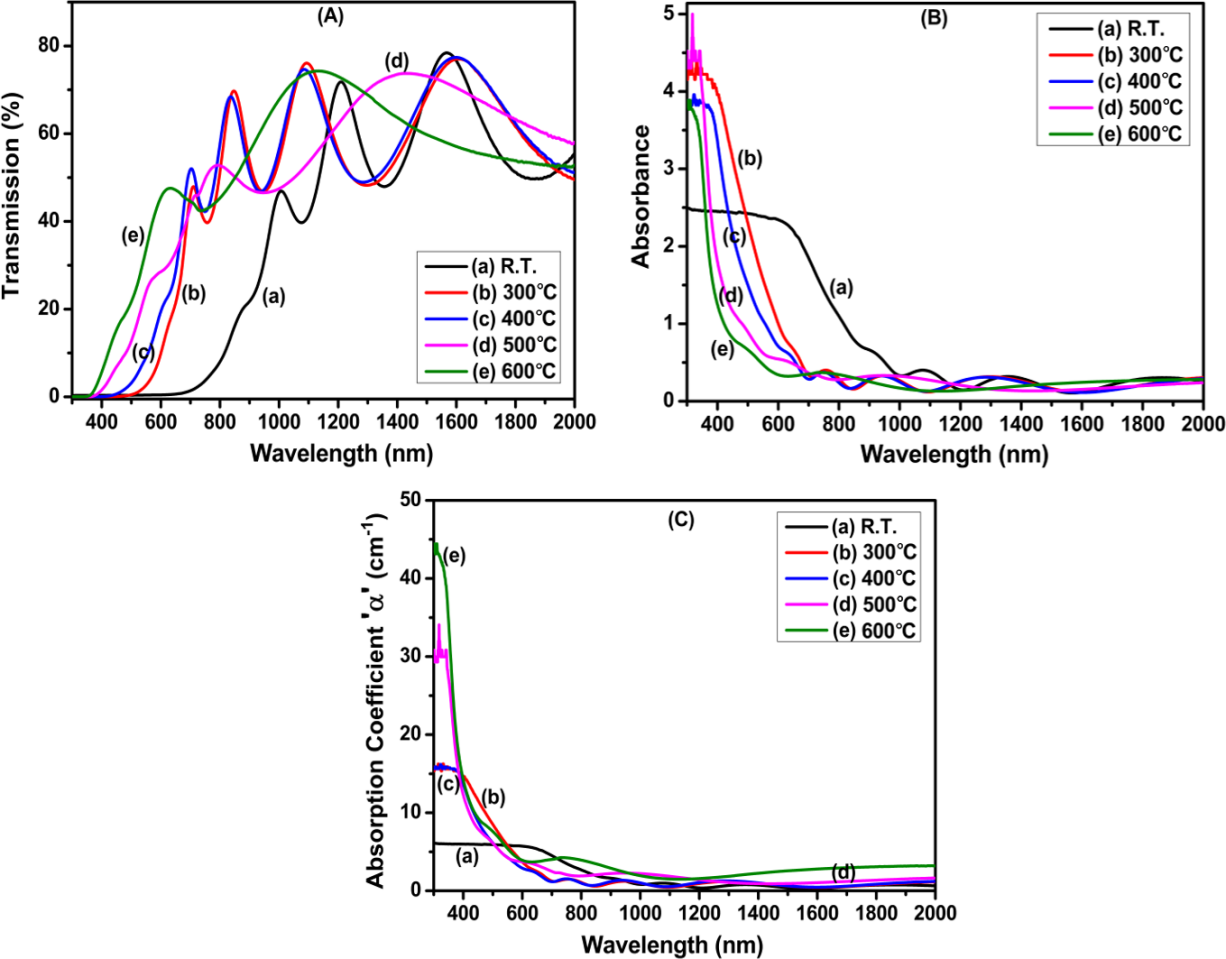
Variation in (A) transmittance, (B) absorbance, and (C) absorption coefficient with wavelength of ZnTe films produced at (a) R.T. and substrate temperatures of (b) 300 °C, (c) 400 °C, (d) 500 °C, and (e) 600 °C.

The transmittance spectra of fabricated ZnTe/Qz films in the wavelength range from 300 to 2000 nm are presented in Figure 5A. A number of interference fringes are observed in the transmittance spectra for all film samples. The interference fringes in the transmittance spectra result from the interference of two beams, one reflected from the surface and the other from the film–substrate interface. The occurrence of interference fringes in the transmittance spectra implies that there is a well-defined boundary between film and substrate and that films with uniform thickness were grown [33]. The number of interference fringes in the transmission spectra is related to the thickness of the deposited film. With increasing substrate temperature, the desorption of atoms increases. For the film deposited at substrate temperatures of 500 and 600 °C, the thickness of deposited films is low compared to that of films deposited at lower substrate temperatures. Because of this, the transmission spectra of the films deposited at these substrate temperatures differ from the others [15,17].

From the transmittance spectra, it has been found that the films deposited at room temperature exhibit transparency in the NIR region only. Whereas the films deposited at higher substrate temperatures exhibits transparency in both visible and NIR regions. Further, it is observed that the average transmittance of the films increases with increasing substrate temperature, which may be because of the higher crystallinity of films with increasing substrate temperature. The enhancement of transmittance in the vis–NIR region with increasing substrate temperature indicates a possible application of these films in optoelectronic devices. The variation in absorbance with wavelength for ZnTe films deposited at different substrate temperatures is presented in Figure 5B. It has been found that the films exhibit maximum absorbance in the visible region, which later decreases with the increase in wavelength. This decrease in absorbance with wavelength may be due to interband electronic transitions between the conduction band and ionized donor levels.

The absorbance was also found to decrease with increasing substrate temperature. This is due to the increase in the transparency of films with increasing substrate temperature. A blue shift in the absorption edge was observed with increasing temperature. The absorption coefficient (α) for the produced films was determined from the transmittance spectra using the relation [34]


[8] $$\alpha =\frac{1}{t}\ln\left(\frac{1}{T}\right),$$

where *T* is the transmittance and *t* represents the thickness of films, which is about 940 ± 0.53 nm, 623 ± 0.16 nm, 563 ± 0.02 nm, 337 ± 0.02 nm, and 200 ± 0.30 nm for films deposited at R.T., 300 °C, 400 °C, 500 °C, and 600 °C, respectively.

Figure 5C shows the absorption coefficient as a function of the wavelength for ZnTe/Qz films at different substrate temperatures. From Figure 5C, it is observed that α has very low values at higher wavelengths (≥600 nm). The absorption coefficient increases very slowly in this region. In contrast, a sharp increase in α is observed in the lower-wavelength (≤600 nm) region. This type of variation in absorption coefficient points toward the existence of both direct and indirect bandgaps in a material. ZnTe films exhibit both direct and indirect transitions [35–36]. Therefore, in this study we have determined both direct and indirect bandgaps.

The Tauc relation was used to determine the optical bandgaps (*E*_g_) of ZnTe/Qz films [34]


**Figure 6:**
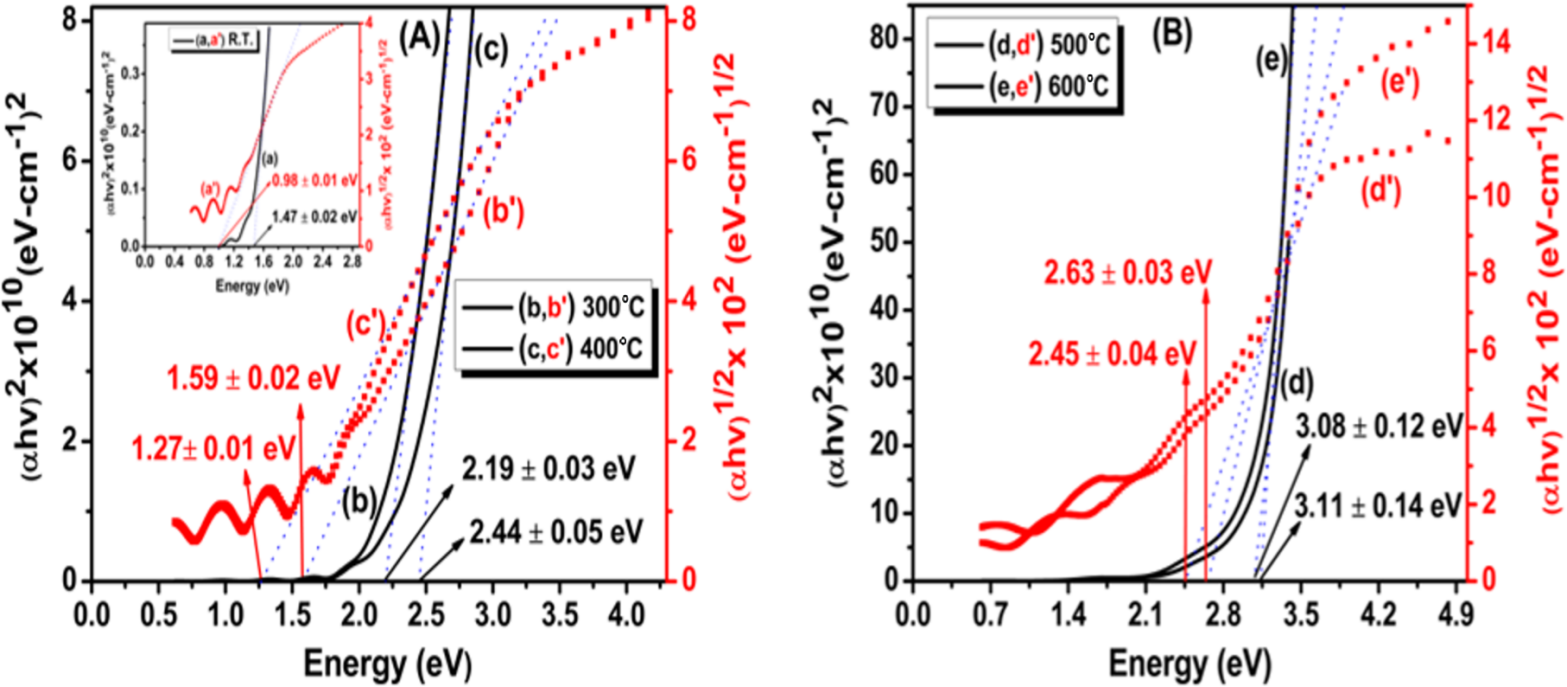
Tauc plots for direct bandgap (left Y-axis scale, a–e) and indirect bandgap (right Y-axis scale, a′–e′) calculation of ZnTe films fabricated at (A) RT (a, a′) (displayed in inset), 300°C (b, b′), 400°C (c, c′); (B) 500°C (d, d′) and 600 °C (e, e′). *R*^2^ = 0.99.

[9] $$\alpha h\nu =C{\left(h\nu -E_{\text{g}}\right)}^{m},$$

where *h* represents the Planck constant, ν is the frequency of the incident light, α is the absorption coefficient, and *C* is an energy-independent constant. The value of the power exponent *m* depends on the type of transition in the films. For a direct allowed transition, *m* takes a value of 1/2. Figure 6 (left Y-axis scale, Figure 6A(a,b,c) and Figure 6B(d,e)) shows the (α*hν*)^2^-vs-*hν* graph for ZnTe films deposited at different substrate temperatures. For obtaining *E*_g_, the linear portion of the graph was extrapolated onto the *x*-axis. The intercept on the *x*-axis gives the optical bandgap value. The direct optical bandgap value for the film deposited at room temperature was found to be 1.47 eV, which is much less than that of the bulk counterpart, which is 2.26 eV. This may be due to the presence of a high density of localized states present near the band edge, which was confirmed by GXRD results. Further, the direct optical bandgap value increases from 2.19 eV at 300 °C to 3.11 eV at 600 °C. The increase in the direct bandgap value with substrate temperature is assigned to the enhancement in crystallinity and decrease in dislocation density in the films [15].

For indirect allowed transitions, *m* = 2. Figure 6 (right Y-axis scale, A(a′, b′, c′) and B(d′, e′)) shows the (α*hν*)^1/2^-vs-hv plot for ZnTe films deposited at different substrate temperatures. The linear portion of the graph was extrapolated to find the bandgap values. The indirect bandgap also increases, from 0.98 eV at R.T. to 2.63 eV at 600 °C, with the rise in substrate temperature. Pal et al. reported changes in direct and indirect bandgap values of ZnTe thin films with varying thickness [35]. However, the changes we observed for direct bandgaps (1.47–3.11 eV) and indirect bandgaps (0.98–2.63 eV) are more significant.

The existence of both direct and indirect bandgaps in a material has implications regarding the efficiency of solar cells. The efficiency of solar cells can be increased if most of the incident photons are absorbed by the absorbing layer and used in charge carrier generation. For this, a large thickness is required in solar cell construction. But thickness has a negative effect on the efficiency because the diffusion length of minority carriers is short for large thickness; thus, not every charge carrier that is generated will participate in conduction. Direct-bandgap materials also have a very short diffusion length of minority carriers, while the diffusion length of minority carriers is very long for the indirect-bandgap materials. Therefore, the best choice is a material having both direct and indirect bandgaps along with some localized states for photon absorption and charge carrier generation [36]. The existence of both direct and indirect bandgaps and their tuneable nature with substrate temperature point toward the application of the fabricated ZnTe films in solar cells.

#### Refractive index

The refractive index is one of the important optical parameters as it is connected to the electronic polarizability, local fields in the semiconducting material, and transmission. The refractive index is related to the transmission of films through the relation [37]


**Figure 7:**
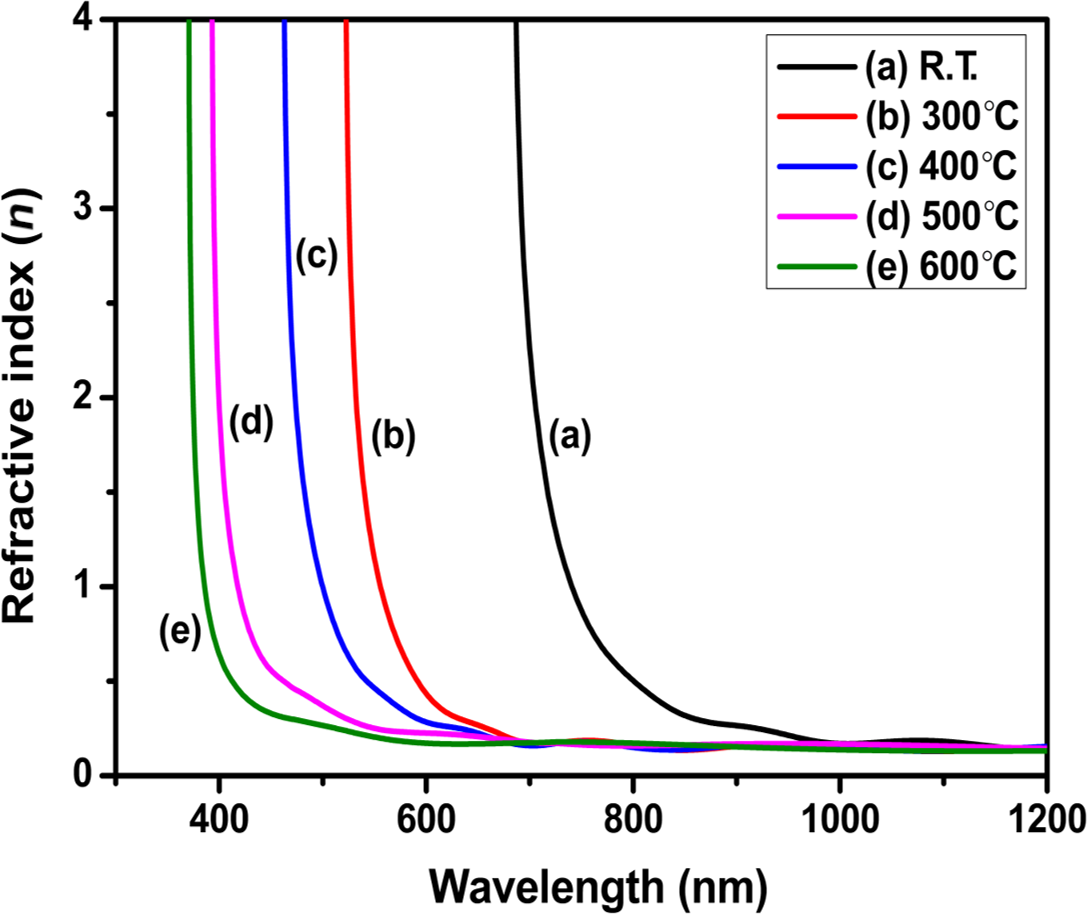
Variation in refractive index (*n*) with wavelength of ZnTe films deposited at (a) R.T. and substrate temperatures of (b) 300 °C, (c) 400 °C, (d) 500 °C, and (e) 600 °C.

[10] $$n=\frac{1}{T}+{\left(\frac{1}{T-1}\right)}^{1/2},$$

where *T* represents the transmission in percent. Figure 7 shows the variation in refractive index with wavelength for films fabricated at various substrate temperatures (R.T.–600 °C). The change in the refractive index shows a normal dispersion behaviour. The refractive index has a high value in the lower-wavelength region. It decreases sharply and becomes nearly constant in the higher-wavelength region. This is because, at lower wavelengths, the absorption capacity of a material is high, which results in a decrease in the speed of light, thus increasing the value of *n*. The refractive index value is also found to decrease with increasing substrate temperature. This may be due to the enhancement in crystallinity and decrement in dislocation density with substrate temperature.

This variation in refractive index value is also associated with the optical bandgap according to the Harve–Vandamme model [38]


[11] $$n^{2}=1+{\left(\frac{A}{E_{\text{g}}+B}\right)}^{2},$$

where *A* (= 13.6 eV) and *B* (= 3.4 eV) are constants, and *E*_g_ represents the direct bandgap. Using this formula, the refractive index has been calculated. The refractive index value decreases from 2.96 at R.T. to 2.31 at 600 °C because of the bandgap increment [39]. The dielectric constant and the dielectric loss of the deposited ZnTe films exhibit a similar pattern and were also found to decrease with increasing substrate temperature (see below).

### Photoluminescence studies

**Figure 8:**
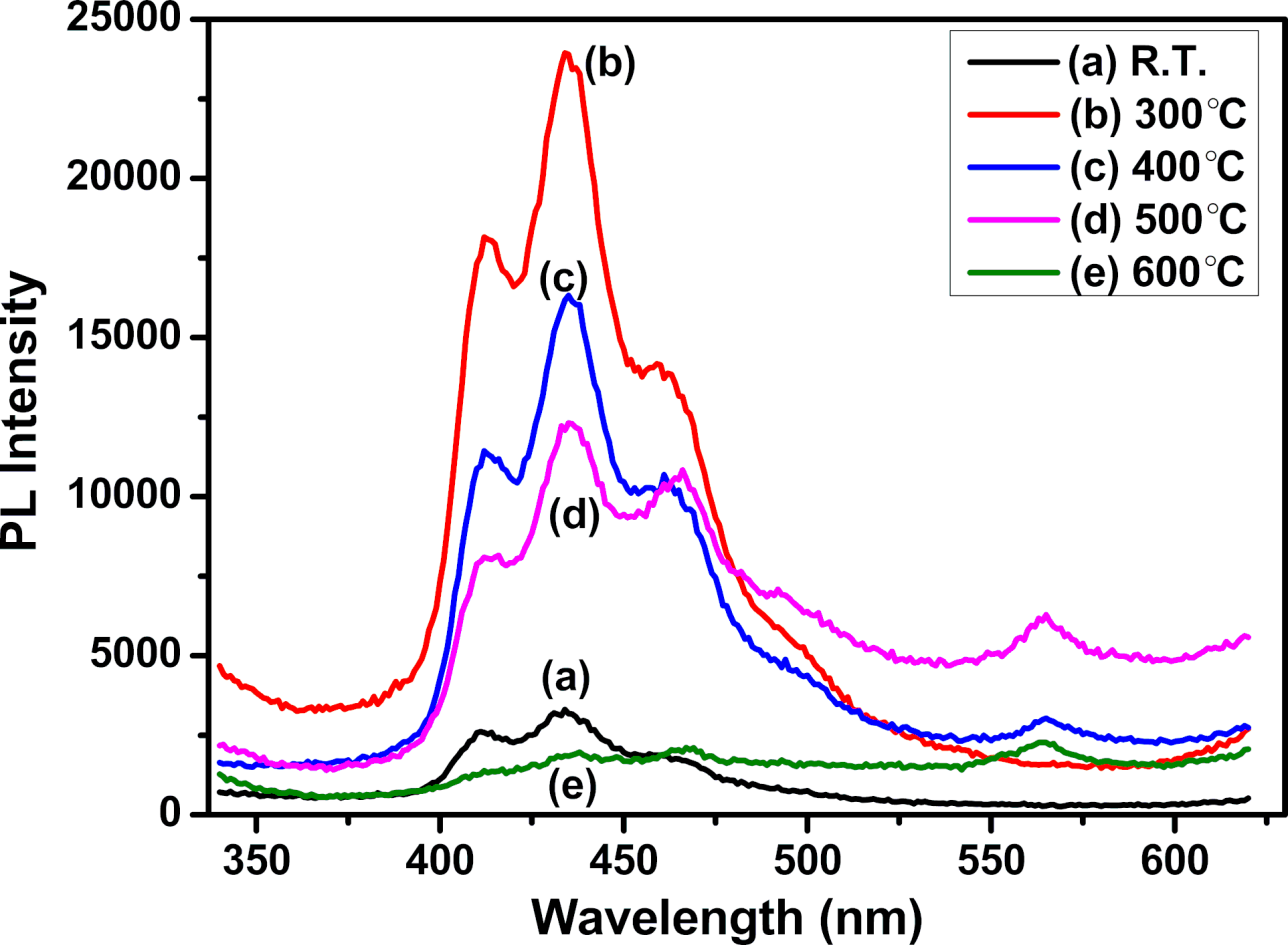
Photoluminescence spectra of ZnTe films deposited at (a) room temperature (R.T.) and substrate temperatures of (b) 300 °C, (c) 400 °C, (d) 500 °C, and (e) 600 °C.

Photoluminescence (PL) studies were carried out to analyse the films’ electronic features. The photoluminescence occurs when a material absorbs energy higher than its bandgap. In a semiconductor, this leads to the creation of a large number of electrons and holes in comparison to their equilibrium concentration. These generated charge carriers recombine after thermal relaxation, and photons with energy lower than the excitation photon energy are emitted. The recombination can occur either from band to band or through impurities and defects present within an energy level inside the forbidden gap. Grain boundaries are responsible for non-radiative recombination processes. For the present analysis, the PL spectra of the ZnTe films were recorded at room temperature using an excitation wavelength of 320 nm from a Xenon arc lamp. Figure 8 shows the PL spectra of ZnTe films deposited at different substrate temperatures. A strong and broad emission spectrum is observed for all film samples in the wavelength range from 420 to 453 nm, with shoulders at 413 and 476 nm. With the increase in substrate temperature (400–600 °C), an additional peak at 563 nm appears. A red shift in the emission peaks is observed at higher substrate temperatures compared to the spectrum of the room-temperature sample. The peaks observed in the emission spectra are accredited to deep and shallow band transitions. The variation in the width of emission peaks may be due to the change in the lattice environment with substrate temperature. The photoluminescence intensity depends on the number of charge carriers that undergo direct band-to-band recombination [40]. The PL intensity decreases with an increase in substrate temperature (300–600 °C). This may be because ZnTe exhibits both direct and indirect bandgaps. The direct bandgap increases with substrate temperature as shown in our bandgap studies. Therefore, the possibility for indirect transition increases with an increase in substrate temperature, which leads to a decrease in PL intensity with substrate temperature. The decrease in PL intensity corroborates our structural and morphological studies, where it was found that both dislocation density and particle density decrease with increasing substrate temperature [40].

### Electrical studies

**Figure 9:**
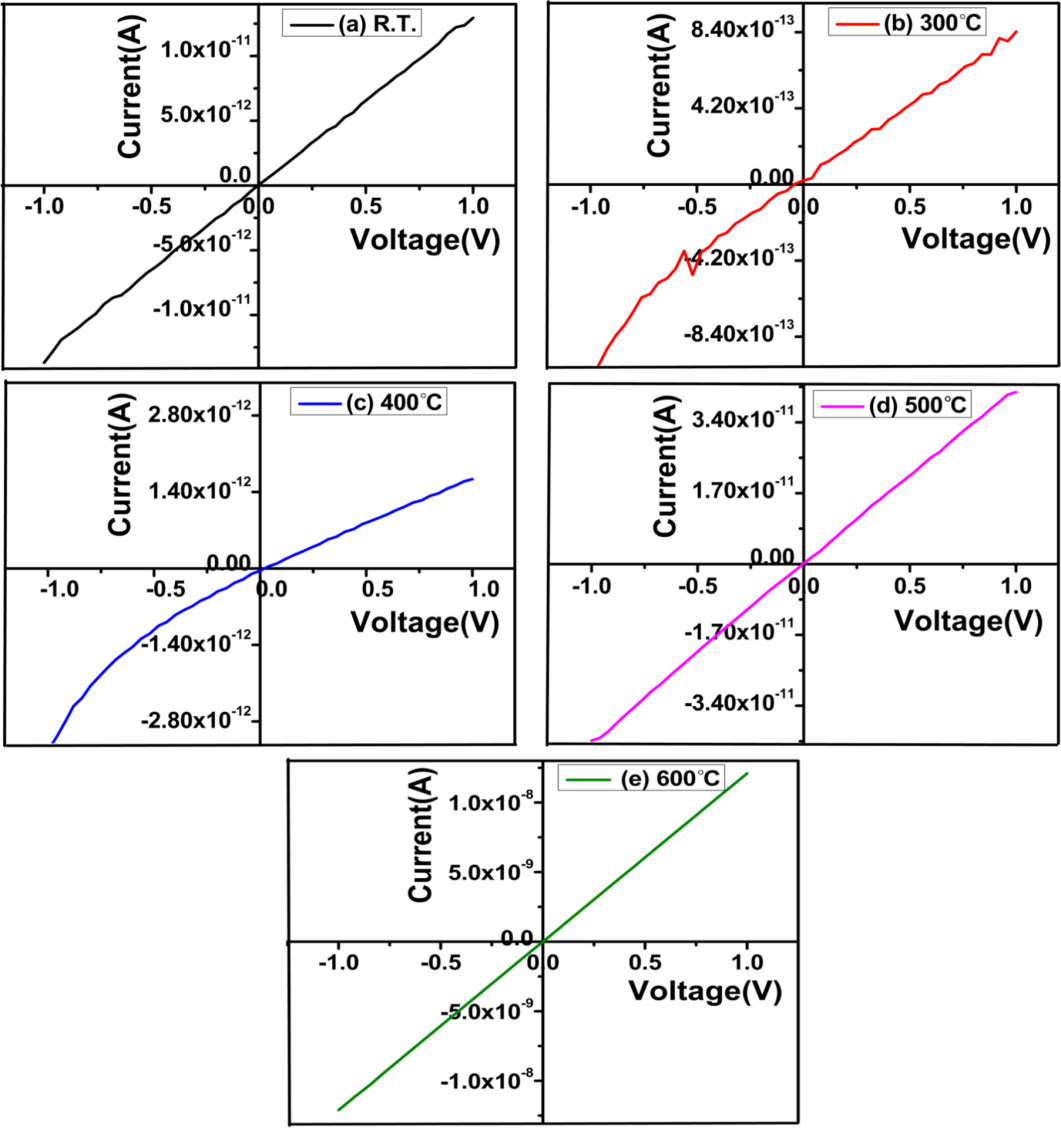
*I*–*V* characteristics of ZnTe films deposited at different substrate temperatures in the range of R.T.–600 °C.

Figure 9 depicts the current–voltage (*I*–*V*) characteristics of ZnTe films deposited at different substrate temperatures. The *I*–*V* characteristics were recorded using a two-probe arrangement provided with a Keithley 4200 SCSA parametric analyser. For this, equally spaced ohmic contacts were made on the surface of the films using silver paste. The characteristics were recorded in the voltage range from −1 V to 1 V. The *I*–*V* characteristics are nearly linear and regular in both positive and negative voltage regions. This points towards a nearly ohmic contact between ZnTe film and quartz substrate, which is necessary for the fabrication of the optoelectronic device.

The conductivity (σ) of films was determined using the relation [41]


[12] $$\sigma =\left(\frac{1}{\rho }\right)=\left(\frac{l}{A\times R}\right),$$

where ρ is the electrical resistivity, *R* is the resistance of the film, *A* is the area of ZnTe film samples (width of film × thickness of film), and *l* corresponds to the distance between the probes (1 cm) while taking the measurement. The measured values of resistance, resistivity, and conductivity are presented in Table 5. The conductivity of the ZnTe films increases (from 1.42 × 10^−8^ to 6.02 × 10^−4^ Ω^−1^·cm^−1^) with increasing substrate temperature (from 300 to 600 °C). The increase in mobility, variation in charge carrier concentration, and enhancement in the crystallinity of the films with increasing substrate temperature might be the possible reason behind the increase in conductivity of films with substrate temperature [42].

**Table 5:** Variation in resistance, resistivity, and conductivity of ZnTe films deposited at various substrate temperatures.

Substrate temperature (°C)	Resistance (Ω)	Resistivity (Ω·cm)	Conductivity (Ω^−1^·cm^−1^)

R.T.	7.63 × 10^10^	7.20 × 10^6^	1.39 × 10^−7^
300	1.13 × 10^12^	7.04 × 10^7^	1.42 × 10^−8^
400	5.20 × 10^11^	2.93 × 10^7^	3.41 × 10^−8^
500	2.35 × 10^10^	7.94 × 10^5^	1.26 × 10^−6^
600	8.25 × 10^7^	1.66 × 10^3^	6.02 × 10^−4^

Grain boundaries have a significant effect on the electric transport properties in polycrystalline films. Grain boundaries are growth process-dependent phenomena and have a large number of charge-trapping centres. Thus, grain boundaries reduce the mobility of charge carriers, and various scattering events take place at grain boundaries such that the conductivity of the films decreases. The number of grain boundaries decreases with an increase in crystallite size. Thus, the increase in crystallinity and decrease in dislocation density with substrate temperature might be the possible reasons for an increase in the conductivity of films. To understand the dependence of the electrical properties of films on different parameters, it is essential to measure the electrical properties of grain boundaries.

## Conclusion

In this work, the effect of substrate temperature on various properties of RF-sputtered ZnTe films was investigated systematically. The structural investigations using GXRD revealed that the films are polycrystalline with cubic zincblende structure. The crystallite size increases (from 37.60 to 68.88 Å) with increasing substrate temperature (300–600 °C). The crystallite size was found to be maximum for the film produced at 600 °C. Optical studies revealed that the average transmittance of ZnTe films increases with substrate temperature. A blue shift in the bandgap was observed with increasing substrate temperature. Taking into account the possibility for indirect transitions, the indirect bandgap for ZnTe films was calculated. Morphological investigation revealed that the roughness increases and the particle density decreases with the increase in substrate temperature. The electrical conductivity was found to increase (from 1.42 × 10^−8^ to 6.06 × 10^−4^ Ω^−1^·cm^−1^) with the increase in substrate temperature (300–600 °C). The observed large bandgap (3.11 eV), high transmittance, large crystallite size (68.88 Å) and high conductivity of the ZnTe film produced at 600 °C indicate a possible application of such films as a buffer layer in solar cells.

## Supporting Information

File 1 Additional data on optical measurements.

## Data Availability

Data generated and analyzed during this study is available from the corresponding author upon reasonable request.
