# Supplementary material for: Tailoring of physical properties of RF-sputtered ZnTe films: role of substrate temperature
Source: Beilstein J Nanotechnol. 2025 Mar 5;16:333–48. doi: 10.3762/bjnano.16.25 (PMC11897647; doi:10.3762/bjnano.16.25)
Supplement: File 1 — Additional data on optical measurements. [file Beilstein_J_Nanotechnol-16-333-s001.pdf]

BEILSTEIN JOURNAL OF NANOTECHNOLOGY

Supporting Information

for

Tailoring of physical properties of RF-sputtered ZnTe films: role of substrate temperature

Kafi Devi, Usha Rani, Arun Kumar, Divya Gupta and Sanjeev Aggarwal

Beilstein J. Nanotechnol. **2025,** *16,* 333–348. doi:10.3762/bjnano.16.25

Additional data on optical measurements

License and Terms: This is a supporting information file under the terms of the Creative Commons Attribution License (https://creativecommons.org/licenses/by/4.0). Please note that the reuse, redistribution and reproduction in particular requires that the author(s) and source are credited and that individual graphics may be subject to special legal provisions.
The license is subject to the *Beilstein Journal of Nanotechnology* terms and conditions: (https://www.beilstein-journals.org/bjnano/terms)

Spectroscopic Ellipsometry

Spectroscopic ellipsometry (SE) is a very useful technique for the determination of thin films' thickness and optical constants because of its non- contact and non-destructive characteristics. In SE, a thin film is illuminated with polarized light, and the reflected polarized light is received in a detector. The change in the polarization after reflection is measured as a function of the wavelength in terms of the two parameters ψ and Δ. The parameter ψ represents the ratio of amplitudes, and Δ represents the change in phase of polarized light. The two parameters are related as

$$\rho = r_s / r_p = \tan\psi \cdot e^{i\Delta},$$

where r_s is the reflection coefficient for s-polarized light, and r_p is the reflection coefficient for p-polarized light. The optical constants of the films are related to these parameters. Based on film structure, an appropriate model is required for fitting the ellipsometry spectra. Here, an optical four-layer model (air/upper roughness layer/ZnTe layer/quartz substrate) as shown in Figure S1 is used.

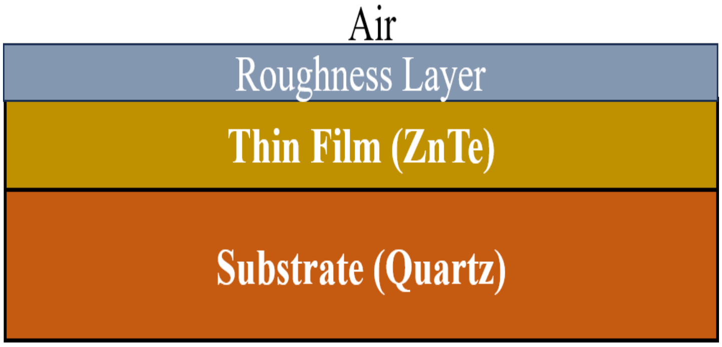

Figure S1: Optical model for deposited ZnTe films consisting of air/upper roughness layer/ZnTe layer/quartz substrate.

The roughness layer in an effective medium approximation and consists of 50% ZnTe and 50% voids. The Tauc–Lorentz dispersion relation is utilized for finding the film thickness. The different fitting parameters are varied using least square regression analysis until a minimum difference between experimental and theoretical spectra is obtained. The experimental and fitted spectra of ψ and Δ for the films deposited at different substrate temperatures are presented in Figure S2. All spectra are well fitted with the theoretical spectra with a mean square error less than 2. The different parameters used for fitting the spectra and the obtained thickness values are given in Table S1, where A is the amplitude, E_0 is the peak transition energy, and C is the broadening of the oscillator.

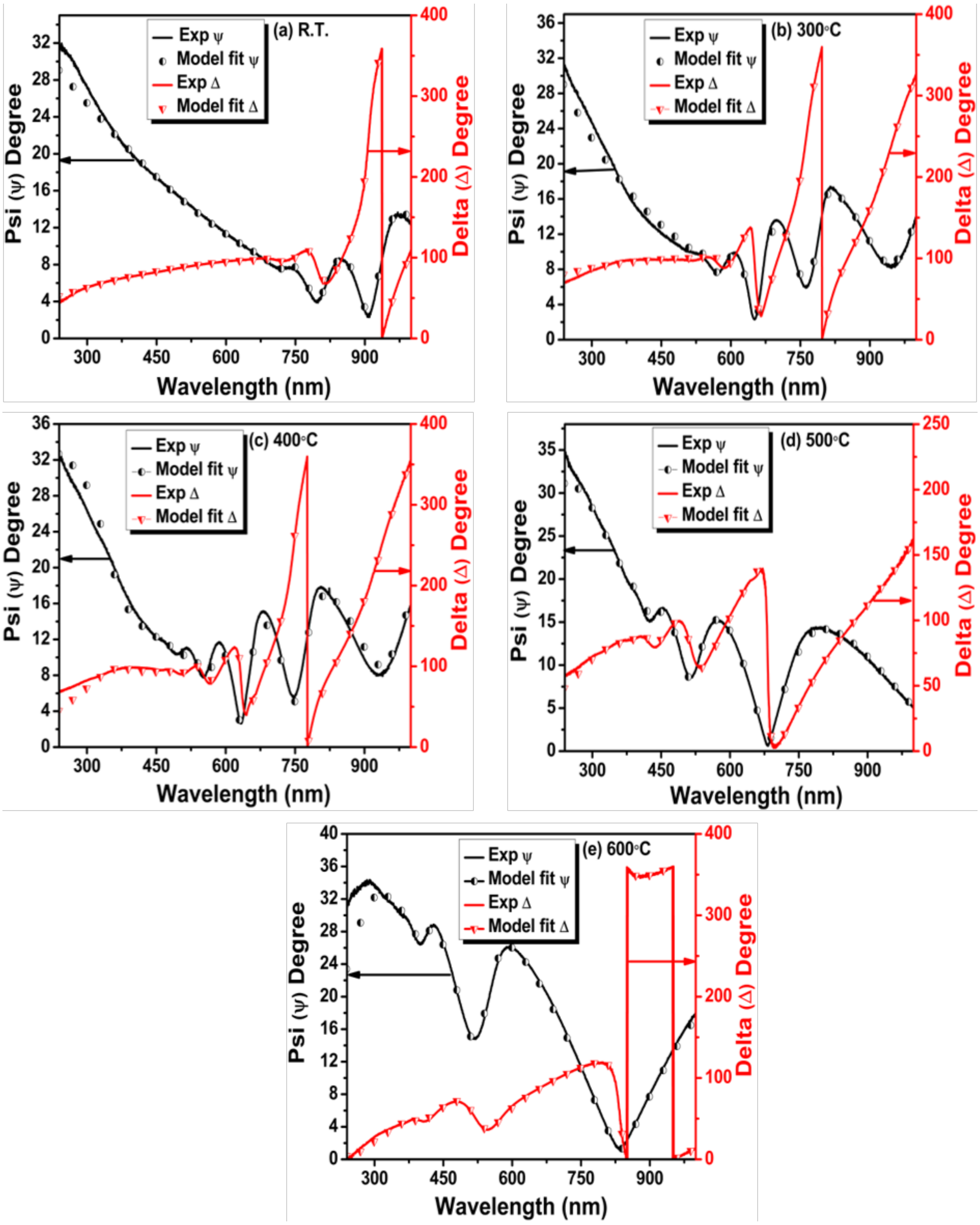

Figure S2: Plots of experimental and simulated (model) SE spectra (ψ and Δ) of ZnTe samples. (a) R.T., (b) 300 °C, (c) 400 °C, (d) 500 °C, and (e) 600 °C.

Table S1: Different fitting parameters used for modeling the simulated data.

Substrate temperature (°C)	A (eV)	E_0 (eV)	C (eV)	Thickness (nm)
R.T.	69.87	3.1170	3.4202	940 ± 0.53
300	113.84	5.7605	5.5627	623 ± 0.16
400	45.67	3.4857	1.0724	563 ± 0.02
500	28.57	4.1079	1.6677	337 ± 0.02
600	126.05	7.2384	7.4807	200 ± 0.30

Extinction coefficient (*k*) and skin depth (δ)

The extinction coefficient gives (*k*) an idea about the interaction of electromagnetic waves with the film; phenomena such as scattering and absorption may occur. The value of the extinction coefficient corresponds to the amount of scattering and absorption of the electromagnetic waves inside the film. The *k* value is obtained from absorption coefficient (α) and wavelength (λ) using the relation [1]

$$k = \frac{\alpha\lambda}{4\pi}.$$

Figure S3A depicts the change in extinction coefficient (*k*) with wavelength (λ) for films grown at different substrate temperatures.

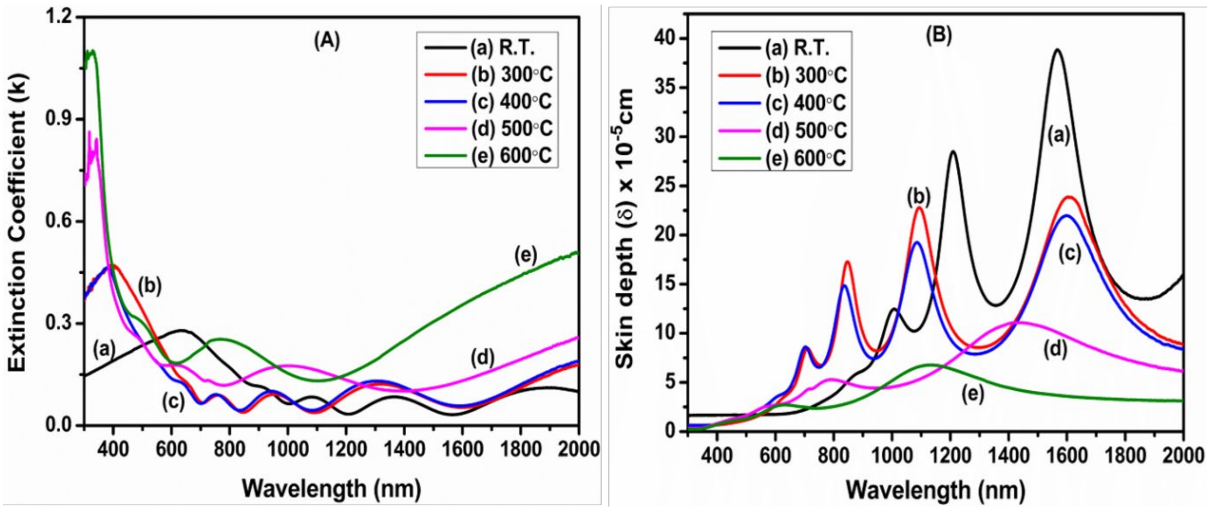

Figure S3: Plots of (A) extinction coefficient and (B) skin depth (δ) of ZnTe films deposited at (a) R.T. and substrate temperatures of (b) 300 °C, (c) 400 °C, (d) 500 °C, and (e) 600 °C.

The extinction coefficient of the films was found to first increase with substrate temperature followed by a gradual decrease in its value with wavelength in the visible region for all samples. The penetration depth gives details about how far electromagnetic waves travel inside the film. The skin depth (δ) represents the film thickness at which the electromagnetic wave intensity reduces to 1/*e* of its value on the surface of the film. The skin depth is inversely related to the photon frequency and conductivity of films. Therefore, high skin depth results from a lower bandgap value [2]. The skin depth is calculated from the absorption coefficient (α) using the relation [2]

$$\delta = \frac{1}{\alpha}.$$

The variation in δ with the wavelength for films grown at different substrate temperatures is shown in Figure S3B. The skin depth value decreases from 1.6 μm at R.T. to 0.3 μm at 600 °C. This is related to the observed increase in the optical bandgap (E_g) value with substrate temperature.

Complex dielectric constants

The dielectric constant is an intrinsic property of a material. It affects the propagation of electromagnetic radiation and is related to the band structure and density of states within the forbidden bandgap. The complex dielectric constant consists of real and imaginary parts [3]

$$\varepsilon = \varepsilon_1 + i\varepsilon_2.$$

The real part of the complex is the dielectric constant ($\varepsilon 1$). It is related to the property of slowing down the speed of light within the material. The imaginary part, also termed dielectric loss (ε_2), describes the absorption of energy from the electric field due to dipole motion. Dielectric constant and dielectric loss were calculated from the refractive index (n) and extinction coefficient (*k*) using the relations [3]

$$\varepsilon_1 = n^2 - k^2,$$

$$\varepsilon_2 = 2nk.$$

The spectral dependence of ε_1 and ε_2 is presented in Figure S4. It exhibits a pattern similar to that of the refractive index because of the very low extinction coefficient. A decrement in the values of ε_1 and ε_2 is observed with increasing substrate temperature. In an amorphous structure, absorption of photon energy through multiple reflections takes place, which results in a high value of dielectric constant compared to that of a crystalline structure. The observed variation in dielectric constant may be due to enhancement in the crystallinity of films with substrate temperature [4].

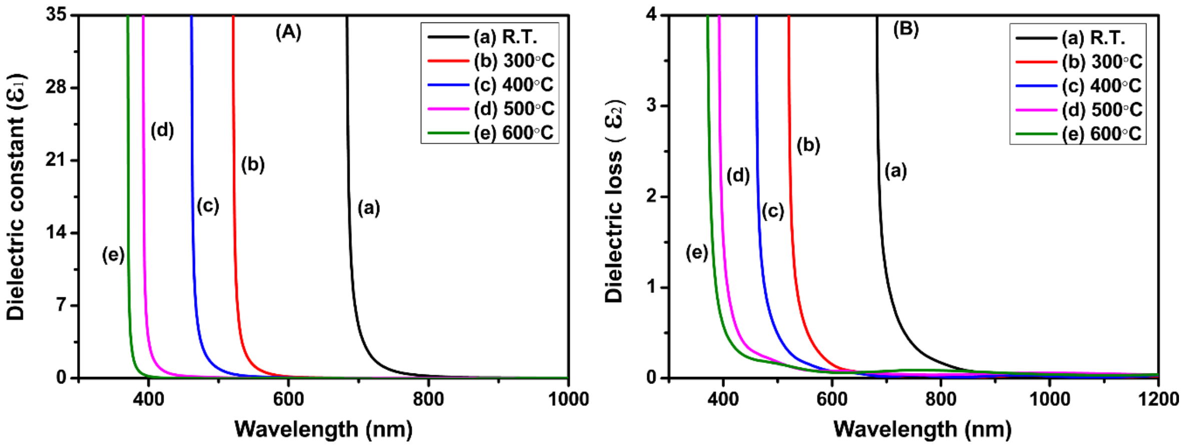

Figure S4: Variation in (A) dielectric constant (ε_1) and (B) dielectric loss (ε_2) with wavelength of ZnTe films deposited at (a) RT and substrate temperatures of (b) 300 °C, (c) 400 °C, (d) 500 °C, and (e) 600 °C.

References

1. Das, S.; Paikaray, Satabdi.; Swain, I.; Senapati, S.; Naik, R. *Surf. Interfaces* **2023,** *42,* 103395. doi:10.1016/j.surfin.2023.103395
2. Dalouji, V. *Heliyon* **2024,** *10,* e37509. doi:10.1016/j.heliyon.2024.e37509
3. Chander, S.; Dhaka, M. S. *J. Mater. Sci.: Mater. Electron.* **2016,** *27,* 11961–11973. doi:10.1007/s10854-016-5343-2
4. Goyal, S.; Chauhan, R. P. *J. Mater. Sci.: Mater. Electron.* **2019,** *30,* 1345–1353. doi:10.1007/s10854-018-0402-5
